# Timing of feeding mitigates metabolic effects of circadian disruption

**DOI:** 10.1038/s44323-026-00093-1

**Published:** 2026-07-27

**Authors:** Nathan J. Skinner, Victoria A. Acosta-Rodríguez, Filipa Rijo-Ferreira, Alexander Tups, Joseph S. Takahashi

**Affiliations:** 1 Department of Neuroscience, Peter O’Donnell Jr. Brain Institute, University of Texas Southwestern Medical Center, Dallas, TX USA; 2 Centre for Neuroendocrinology, Department of Physiology, Faculty of Biomedical Sciences, University of Otago, Dunedin, New Zealand; 3 Howard Hughes Medical Institute, University of Texas Southwestern Medical Center, Dallas, TX USA; 4 Present Address: Circadian Biology of Aging Unit, Translational Gerontology Branch, National Institute on Aging, National Institutes of Health, Baltimore, MD USA; 5 Present Address: Berkeley Public Health, Molecular and Cell Biology Department, University of California, Berkeley, CA USA

**Keywords:** Neuroscience, Physiology

## Abstract

Disrupted light-dark cycles and mistimed feeding are recognized metabolic stressors. In this study, we investigated the independent and combined effects of a 12:12 light:dark cycle, advanced by 6 h every 6 days (Chronic Disruption; CD), and time-restricted feeding on the metabolic health of male C57BL/6J mice. *Ad libitum* mice under the CD cycle gained significantly more body weight and body fat compared to animals under a control light cycle (LD), despite similar caloric intake and wheel running activity. Using specialized equipment to dissociate light cues from nutrient access, we demonstrate that light-cycle disruption induces metabolic dysfunction specifically through fragmented and misaligned eating patterns. Crucially, restricting food access to a 12-h window synchronized with the shifting dark phase abrogated the weight gain and adiposity observed in the *ad libitum* group. Conversely, shifting the feeding window under a stable light cycle significantly reduced food intake and weight gain, revealing that feeding regularity is a primary determinant of energy balance independent of light-cycle stability. Overall, while the light-dark cycle is the dominant zeitgeber for activity, the temporal consolidation of food intake is the primary driver of metabolic alignment, offering a potential intervention for managing metabolic risk in shift workers.

## Introduction

Disruptions in light exposure patterns, particularly artificial light at night, are increasingly recognized as a significant environmental stressor and a critical risk factor for the development of metabolic disorders. With the invention of artificial lighting in modern society came the ability to fundamentally alter the natural light/dark cycle that has shaped mammalian physiology for millennia^1^. This alteration has profound implications for the circadian system, an intricate network of central and peripheral molecular clocks that orchestrate the timing of a vast array of physiological and behavioral processes, including sleep-wake cycles, hormone secretion, body temperature regulation, and metabolism^2^. The circadian system is highly sensitive to light^3,4^, particularly its intensity, wavelength, and timing, with the suprachiasmatic nucleus (SCN) in the hypothalamus serving as the primary pacemaker, synchronizing peripheral clocks throughout the body to the external environment^5^. Disruptions to the normally robust light-dark cycle can severely impair the proper functioning of the SCN and its downstream signaling pathways, leading to a cascade of detrimental metabolic consequences^6^. Prior research has shown that chronic light cycle disruptions, either in shift work or jetlag mimicking protocols, have a wide array of metabolic consequences. Glucose metabolism has been shown to be dysregulated under lighting disruptions^7–10^, leading to varying negative effects on glucose and insulin tolerance. Hepatic lipid storage and plasma triglycerides are increased under light disruptions^11^, and lipid metabolism is impaired by chronic jetlag^10^. Motivation for food intake has been shown to be significantly reduced, suggesting a disruption of central reward pathways^12^. Underlying these changes are dramatic changes in circadian clock genes and clock outputs in various tissues^13–17^.

Beyond light information, the timing of food intake has emerged as another crucial environmental cue, playing a vital role in entraining peripheral circadian clocks and exerting a substantial influence on metabolic health outcomes^18,19^. While the SCN is primarily synchronized by light, peripheral oscillators in organs like the liver, pancreas, and adipose tissue are also highly responsive to meal timing and nutrient availability^20–22^. Regular and appropriately timed food intake reinforces circadian rhythms within these metabolic tissues, optimizing nutrient processing and energy homeostasis^23^. Conversely, mistimed eating patterns can disrupt the synchrony between central and peripheral clocks, contributing to metabolic dysfunction independent of, or in concert with, aberrant light exposure^21^^,^^22^^,^^24^. Alterations to lighting conditions in laboratory animal housing coincide with changes in food consumption patterns, leading to internal desynchronization^25–27^. For humans in particular, later meal timing, food consumption closer to bedtime, or irregular food timing termed “eating jetlag”, is associated with increased body weight gain and increased BMI^28,29^. Chronic misalignment between the internal circadian clock and external cues, common in shift work, jetlag, and social jetlag, significantly increases the risk of developing metabolic disorders such as type II diabetes, obesity, and cardiovascular diseases^30–34^. Epidemiological studies consistently demonstrate a strong association between these circadian disruptions and adverse metabolic profiles, highlighting the clinical significance of maintaining robust circadian alignment.

While the long-term health consequences of incorrect circadian timing are well-documented, the underlying mechanisms by which this occurs remain an area of active investigation. It is increasingly evident that in individuals experiencing chronic circadian disruption, the intricate internal synchrony of their cyclic oscillators becomes progressively misaligned, not only with external cues but also with one another^35^. This internal desynchrony manifests as a state in which critical behavioral, metabolic, hormonal, and neuronal variables, which are normally tightly coordinated in their rhythmic oscillations, begin to cycle out of phase. We have shown previously that this temporal misalignment at the systemic level disrupts key metabolic signaling pathways within the brain and contributes to metabolic impairments^36^.

While previous studies have established that circadian disruption induces metabolic strain, the confounding of light-driven activity shifts and fragmented feeding behavior has made it difficult to isolate the primary driver of weight gain. By utilizing specialized feeding systems, our study provides a clean dissociation between light cycle disruption and feeding regularity, allowing us to evaluate whether nutrient timing alone can override the metabolic penalty of a shifting environment. Initially, we assess the physiological and behavioral impact of chronic lighting disruptions and attempt to rescue negative effects by time-restricted feeding. We then evaluate the effect of independently disrupting the light and food zeitgeber and determine the effect that this has on energy balance. Our main result indicates that the temporal restriction of feeding can mitigate the excessive adiposity and weight gain caused by chronic light disruption. Understanding this might have a positive effect in improving the health of shift workers and individuals experiencing regular circadian disruptions.

## Results

### Mismatch of food intake and light conditions disrupt mice behavior

**Fig. 1:**
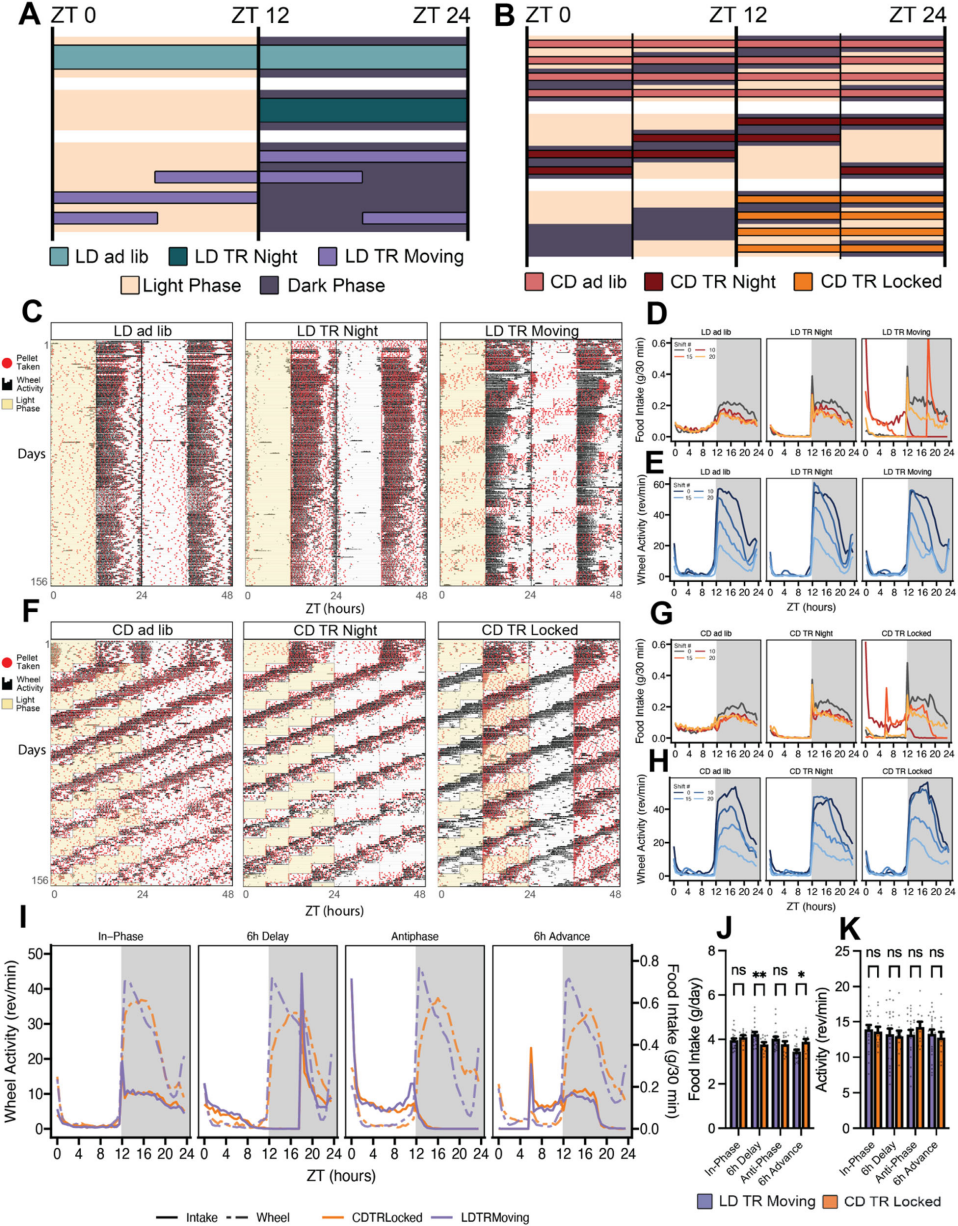
Diagrammatic representation of light cycle groups. Light cycle and feeding paradigms. **A** Mice under the control light cycle (LD) were subjected to a 12:12 light/dark cycle for the entirety of the experiment. Light/dark shade indicates light cycle, coloured bars indicate food access, white horizontal bars show separation between feeding paradigms. **B** Mice under the chronic disruption (CD) light cycle had their 12:12 light/dark cycle advanced by 6 h, every 6 days. **C** Representative double plotted actograms for LD Cycle and **F** CD Cycle where black bars indicate running wheel activity, orange dots indicate when a pellet was taken, and shaded yellow indicates when lights were on. **D**, **G** Average food intake and **E**, **H** running wheel activity of animals, averaged across the 6 days of selected phase shifts (Shift 0,10,15,20). **I** Average food intake (solid line) and running wheel activity (dashed line) for animals in the LD TR Moving and CD TR Locked groups, across the difference phase differences of restricted food intake window to light/dark cycle, averaged across the entire experiment. **J** Average food intake and **K** wheel activity for each phase difference between food intake window and light dark cycle. Despite the differences in phase between food intake restriction and light/dark cycle, there are no observed difference in activity or food intake amounts. Data shows mean ± SEM; *n* = 12–24.

Under control conditions, food intake and running wheel activity in nocturnal mice is consolidated to the dark or active phase, as seen in the LD *ad libitum* fed profiles (Fig. 1C–E). Food intake follows a diurnal pattern that begins to rise at the onset of the dark phase (ZT12), peaking around ZT18–ZT20 before gradually tapering off toward the light phase (ZT0). Under a shifting light cycle, as is the case in CD *ad libitum* fed mice, this behavioral organization is visibly degraded. Actograms reveal a consistent shift in both the wheel and food intake activity of the mice under the CD cycle, as the light schedule advances by 6 h every 6 days (Fig. 1F). CD a*d libitum* fed animals exhibit a noticeably flatter and more fragmented feeding rhythm, with reduced peak amplitude and food intake spread sporadically across the 24-h cycle, particularly as the experiment progresses (Fig. 1G). Running wheel activity, however, remains similar to LD *ad libitum* fed mice, in all CD cycle groups (Fig. 1H). This is likely due to a masking effect of light, which exerts a potent suppressive influence on locomotor activity in nocturnal rodents, effectively anchoring wheel-running to the dark period regardless of the shifting light schedule or feeding status^37^. This phenomenon is visually evident in the CD actograms and wheel profiles, where activity shifts in lockstep with the advancing light cycle, maintaining its exclusivity to the nocturnal period, despite the fragmentation seen in CD *ad libitum* feeding patterns.

In contrast, the TR Night groups are forced into nocturnal synchronization by the restriction of their feeding window. In both LD and CD cycles, these animals exhibit a rigid, high-amplitude feeding block, contained mostly within the ZT12–ZT24 dark phase. The transition into the dark phase is marked by a sharp, vertical increase in intake that quickly stabilizes into a sustained peak. This sharp onset suggests that the restriction during the day drives hunger, leading to a compensatory rebound feeding effect as soon as the food becomes available. For the purposes of this study, we define alignment as this functional synchronization of nutrient intake with the external light/dark cycle and the resulting masked behavioral activity, acknowledging that molecular entrainment of the central pacemaker may lag behind these rapid environmental shifts.

To assess the differential effect of disrupting either the light zeitgeber or the food zeitgeber, we devised two opposing paradigms: LD TR Moving and CD TR Locked. In the LD TR Moving group, food was restricted to a 12 h window that shifted by 6 h every 6 days, in concert with the light schedule of the CD cycle. Conversely, the CD TR Locked group maintained a fixed 12 h window of food intake regardless of how the light cycle shifted through the CD cycle. Both paradigms resulted in a state of desynchrony between the timing of food intake and the light/dark phase, as visualized by the staircase pattern of LD TR Moving feeding across the actogram (Fig. 1C) and the fixed food intake pattern in CD TR Locked actogram (Fig. 1F).

Visually, the LD TR Moving and CD TR Locked food intake profiles are characterized by high-amplitude peaks that shift across the ZT axis over the course of the experiment (Fig. 1D, G). These forced consumption windows often begin with a sharp feeding spike immediately upon the window opening. The relationship of the food intake window to the light cycle did not appear to alter the running wheel activity (Fig. 1I). Whether feeding was in-phase, antiphase, or staggered by a 6-h delay or advance, physical activity remained stubbornly anchored to the dark phase. This highlights a profound decoupling of the two primary *zeitgebers*, where the masking effect of light maintains a rigid nocturnal activity rhythm even as the feeding schedule cycles through the entire 24-h day. Two-way ANOVA revealed no effect of light cycle on the amount of food intake (*F* (1, 34) = 0.058, *p* = 0.8096) or the amount of running wheel activity (*F* (1, 34) = 0.00066, *p* = 0.975) at different phases of desynchrony (Fig. 1J, K). Post-hoc analysis revealed that at the 6 h delay and 6 h advance, there was a mirroring of food intake difference between LD TR Locked and CD TR Moving (*p* = 0.009, *p* = 0.118, respectively).

### Lighting disruptions cause weight gain and fat accumulation

**Fig. 2:**
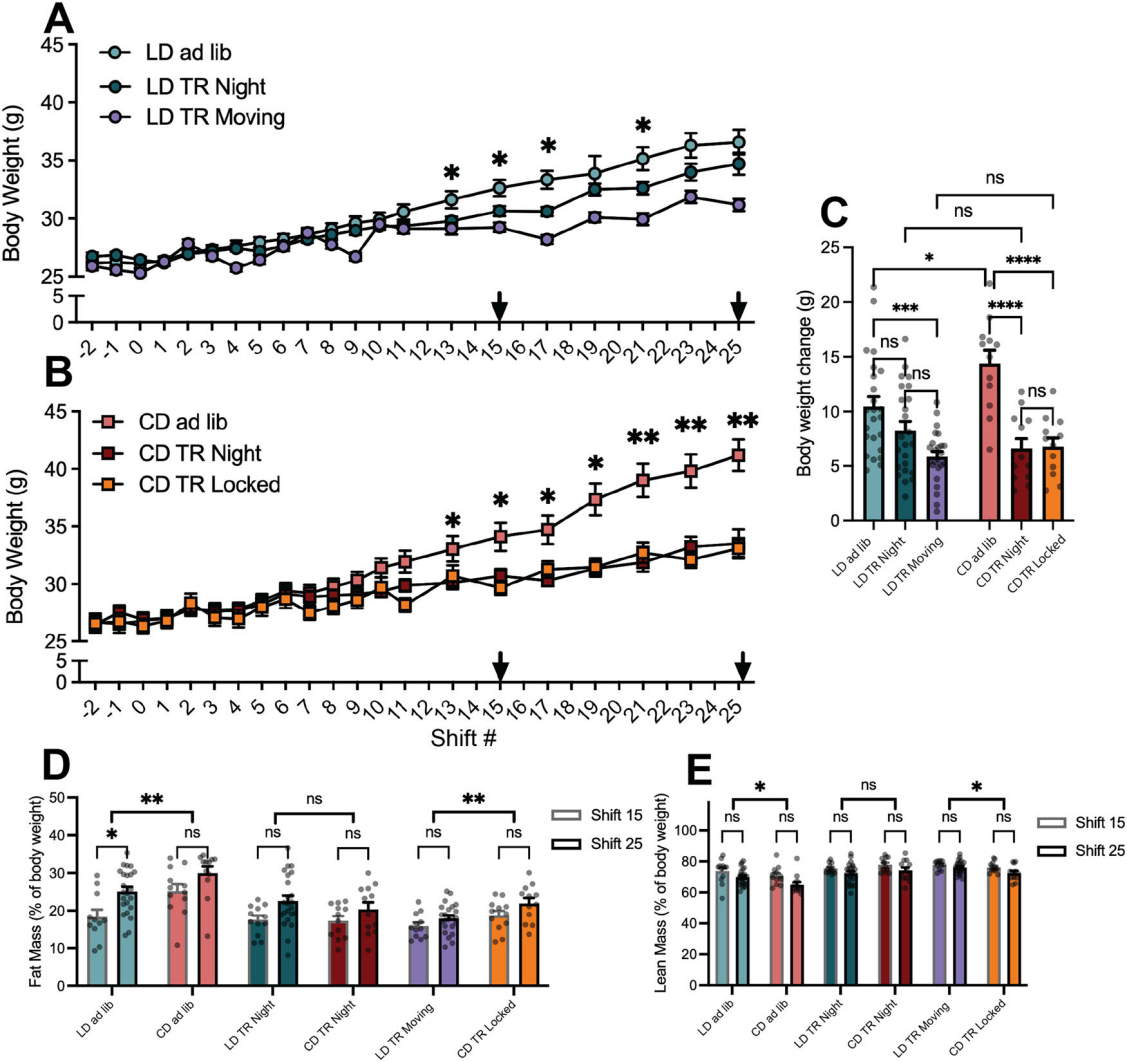
Body weight and composition over the course of the experiment. **A** Body weight trajectory for animals under LD Cycle, fed either *ad libitum*, restricted to the dark phase (LD TR Night) or fed during a shifting 12 h window (LD TR Moving). **B** Body weight trajectory for animals under CD Cycle, fed either *ad libitum*, restricted to the dark phase (CD TR Night) or a locked 12 hr window of feeding (CD TR Locked). **C** Percentage increase in bodyweight from baseline. **D** Fat mass as a percentage of bodyweight for animals, recorded following the 15th phase shift, and at termination. **E** Lean mass as a percentage of bodyweight, recorded following the 15th phase shift, and at termination. Data shows mean ± SEM; *n* = 12–24; ^*^P < 0.05, ^**^P < 0.01, ^***^P < 0.001, ^****^P < 0.0001, n.s not significant.

To explore the physiological effects of prolonged light disturbances and various feeding schedules, we examined changes in body weight and body composition. Animals were weighed every 6 days, one light cycle prior to a phase advance for animals under the CD cycle. We analyzed the longitudinal effects of light cycle and feeding schedule on body weight using a three-way Type III ANOVA. While there were no significant main effects for feeding paradigm (*χ*^2^ (2) = 0.58, *p* = 0.746) or light cycle (*χ*^2^ (1) = 0.29, *p* = 0.589), we observed a highly significant three-way shift *x* feeding *x* light cycle interaction (*χ*^2^ (40) = 184.86, *p* < 0.001), indicating that the impact of circadian disruption on body weight was dependent upon both the specific feeding regimen and the cumulative duration of the intervention. Post-hoc pairwise comparisons revealed that during the initial phase of the study (shifts 1–6), body weight remained comparable across all feeding groups in both LD and CD cycles (*p* > 0.05 for all comparisons; Fig. 2A, B). However, significant divergences emerged as the experiment progressed. CD *ad libitum* mice began showing significantly greater weight gain compared to the CD TR Night group starting at shift 15 (*p* = 0.026), a difference that became increasingly pronounced by the end of the study at Shift 21 (*p* < 0.001). In the LD cycle, a similar divergence was observed, with LD *ad libitum* mice gaining significantly more than the LD TR Night group starting at Shift 15 (*p* = 0.039). By the conclusion of the study (Shift 21), CD *ad libitum* mice had the largest weight gain divergence compared to their restricted feeding counterparts (*p* < 0.001). Analysis of total weight gain from beginning to end confirmed a significant effect of experimental feeding paradigm (*F*(2, 102) = 25.26, *p* < 0.0001) and a significant interaction between feeding and light cycle (*F*(2, 102) = 4.71, *p* = 0.011), though the main effect of light cycle alone was non-significant (*F*(1, 102) = 2.08, *p* = 0.152; Fig. 2C). Under the standard LD cycle, all mice gained weight, with LD *ad libitum* mice gaining significantly more than the LD TR Moving group (*p* = 0.0003). The CD *ad libitum* group exhibited the most pronounced weight gain, diverging significantly from both the LD TR Night (*p* < 0.0001) and LD TR Locked groups (*p* < 0.0001). Most importantly, our data show that light cycle disruption independently exacerbates weight gain when food is available *ad libitum*: CD *ad libitum* mice gained significantly more weight than LD *ad libitum* mice (*p* = 0.0287). Crucially, TR feeding provided a rescue from these effects; CD TR Night and CD TR Locked mice maintained body weight trajectories that were statistically indistinguishable from their LD TR counterparts (*p* = 0.675 and *p* = 0.848, respectively).

Body composition was assessed at two time points using MRI-based technology, providing a longitudinal assessment of both fat (Fig. 2D) and lean mass (Fig. 2E). For fat mass, a three-way ANOVA revealed significant main effects for shift (*χ*^2^(1) = 132.55, *p* < 0.0001), feeding (*χ*^2^ (2) = 15.08, *p* = 0.0005), and light cycle (*χ*^2^ (1) = 6.84, *p* = 0.0089). These were further characterized by significant interactions between shift *x* feeding (*p* < 0.0001) and feeding *x* light cycle (*p* = 0.022), highlighting that the metabolic impact of light cycle disruption is heavily dependent on the feeding paradigm.

Within the CD cycle, post-hoc pairwise comparisons revealed that CD *ad libitum* animals accumulated significantly more fat mass than both CD TR Night (*p* = 0.0009) and CD TR Locked (*p* = 0.002) groups by the 15th phase shift. This divergence widened by the end of the experiment (shift 25), where CD *ad libitum* mice exhibited a 5.45 ± 1.1% greater fat mass than the CD TR Night group (*p* < 0.0001). In contrast, no significant differences in fat mass were observed between feeding groups under the LD cycle at the 15th shift (*p* > 0.05); however, by the final time point, LD *ad libitum* mice had 3.82 ± 0.78% more fat mass than the TR Moving group (*p* < 0.0001), though they remained comparable to LD TR Night animals (*p* = 0.077). When comparing LD and CD cycle directly, *ad libitum* fed animals had an effect of both light cycle (*F* (1, 54) = 11.96, *p* = 0.0011) and shift (*F* (1, 54) = 11.46, *p* = 0.0013), with post-hoc analysis revealing a difference of 6.90 ± 2.58% (*p* = 0.394) at the 15th shift, and a difference of 4.85 ± 2.20% (*p* = 0.935) at the 25th shift, between CD *ad libitum* and LD *ad libitum* mice.

Lean mass analysis revealed a significant main effect of feeding (*χ*^2^ (2) = 11.97, *p* = 0.0025) and shift (*χ*^2^ (1) = 6.94, *p* = 0.0084), though no significant interactions involving light cycle were observed. Interestingly, CD *ad libitum* mice maintained higher lean mass compared to CD TR Night (*p* = 0.036) and TR Locked (*p* = 0.004) groups within the CD cycle. Conversely, in the LD cycle, significant differences in lean mass only emerged at shift 25 between the LD *ad libitum* and LD TR Moving groups (*p* = 0.023), with no significant difference observed between LD *ad libitum* and LD TR Night animals.

### Lighting disruptions and feeding paradigms differentially affect the timing, amount, and regularity of food intake

**Fig. 3:**
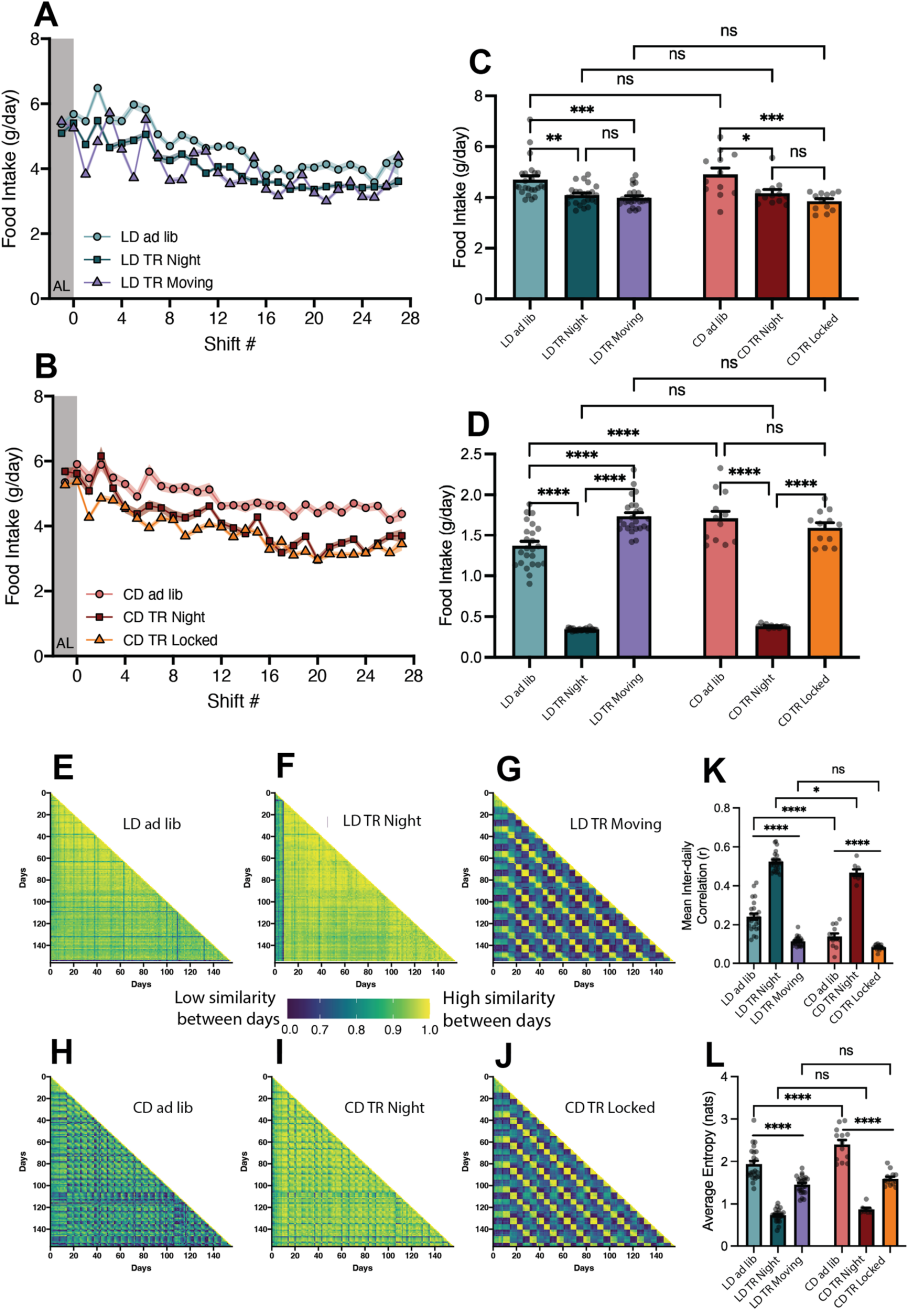
Food intake patterns and stability over the course of the experiment. Daily food intake for animals under the **A** LD Cycle, and **B** CD Cycle**. C** Total food intake for animals under the. **D** Food intake during the light phase (ZT0 – ZT12) as a percentage of total food intake, in animals under. **E**–**G** LD Cycle and **H**–**J** CD Cycle animals autocorrelation plots, showing the similarity of food intake rhythms from all days with all other days. In this heatmap, a high value of 1 (yellow) indicates high similarity in rhythmic food intake between days, and a value of 0 (dark blue) indicates low similarity in rhythmic food intake between days. **K** Mean inter-daily correlation across the entire experiment, calculated from the autocorrelation plots. Both TR Night groups show the strongest inter-daily correlation. LD *ad libitum* mice show significantly stronger inter-daily correlation in comparison to CD *ad libitum* mice. **L** Average entropy calculation across the entire experiment. Entropy is calculated as an indicator of the fragmentation and unpredictability of food intake rhythms. CD *ad libitum* animals have increased entropy, indicating higher variability in food intake rhythms in comparison to LD *ad libitum*. Data shows mean ± SEM; *n* = 12–24; ^*^P < 0.05, ^**^P < 0.01, ^***^P < 0.001, ^****^P < 0.0001, n.s not significant.

To determine if the observed changes in body weight and composition were driven by total caloric intake or altered feeding patterns, we assessed the amount of and timing of food intake. During the initial phase of the study (days 0–11), daily intake was comparable across all groups (Fig. 3A, B). We found a highly significant three-way day *x* feeding *x* light cycle interaction (*χ*^2^ (324) = 1445.83, *p* < 0.001), indicating that the trajectory of food intake was shaped by the combination of all factors over time. Beginning at Day 12, *ad libitum* mice in both light cycle conditions began consuming significantly more food than their TR counterparts (*p* < 0.0001). Crucially, total daily intake did not differ between LD and CD *ad libitum* mice, nor between LD and CD TR mice, throughout the experiment. We then analyzed the volume of longitudinal food intake using a two-way ANOVA (Fig. 3C). We found a significant effect of feeding paradigm (*F* (2, 102) = 21.97, *p* < 0.000), but no effect of light cycle (*F* (1, 102) = 0.1745, *p* = 0.6771) or interaction between the two (*F* (2, 102) = 0.8046, *p* = 0.4501).

We next quantified average light-phase consumption to identify shifts in the timing of intake (Fig. 3D). A two-way ANOVA revealed a significant feeding and light cycle interaction (*F* (2, 102) = 11.19, *p* < 0.0001), as well as a significant effect of feeding paradigm (*F* (2, 102) = 390.7, *p* < 0.0001). Post-hoc comparisons showed that CD *ad libitum* mice consumed nearly 30% of their total daily intake during the light phase, which was significantly more than LD *ad libitum* controls (*p* < 0.0001), who consumed less than 10% during the same period. Conversely, CD TR Night feeding effectively shielded the mice from this disruption. Daytime intake in TR Night groups was minimal (~7%) and did not differ between LD and CD conditions (*p* = 0.813), demonstrating that time-restricted feeding successfully consolidated intake to the dark phase despite environmental light-cycle shifts.

To assess how these paradigms impacted the day-to-day regularity of feeding behavior, we compared the longitudinal patterns of daily intake within each group using autocorrelation analysis (Fig. 3E–J). Between *ad libitum* fed animals, autocorrelation plots show lower correlation in CD cycle animals compared with LD cycle. Specifically, while LD *ad libitum* mice maintain a visible, albeit modest, rhythmic consistency across the duration of the study, the CD *ad libitum* plot is characterized by a noisier distribution and a more rapid decay in correlation. In stark contrast, the LD TR Night and CD TR Night groups show exceptionally high and stable correlation values, visualized as a dense, high-heat diagonal that persists throughout the experiment, regardless of the light cycle. The LD TR Moving and CD TR Locked paradigms produce distinct, periodic “staircase” patterns in the autocorrelation matrices, which directly map to the 6-h phase shifts imposed every 6 days. This confirms that while the shifting schedules force a structural reorganization of behavior, mice are forced to maintain a degree of internal rhythmicity that is entirely absent in the fragmented patterns of the CD *ad libitum* group.

We quantified the consistency of the feeding rhythm by calculating the mean correlation (*r*) of each day’s intake pattern with all other days for every subject (Fig. 3K). A two-way ANOVA revealed significant main effects for both feeding paradigm (*F*(2, 100) = 438.3, *p* < 0.0001) and light cycle (*F*(1, 100) = 33.48, *p* < 0.0001), with a significant interaction between the two (*F*(2, 100) = 4.07, *p* = 0.020). CD *ad libitum* mice showed the lowest inter-daily stability, indicating a progressive degradation of rhythmic consistency under light disruption. Post-hoc pairwise comparisons revealed that the impact of light cycle disruption on behavioral stability varied significantly across feeding regimens. Within the *ad libitum* groups, when comparing LD and CD cycle mice, there was a dramatic reduction in inter-daily stability, with LD mice exhibiting significantly higher mean correlation values than their CD counterparts (*p* < 0.0001).

The TR Night groups maintained the highest overall levels of stability; however, they were not entirely immune to the effects of light disruption. LD TR Night mice showed significantly higher correlation than CD TR Night mice (*p* = 0.0273), though both remained substantially more stable than any other experimental group. In contrast, the TR Moving/Locked paradigms were the least different between the light cycle paradigms; no significant difference in stability was observed between the LD and CD conditions for these groups (*p* = 0.1651).

Finally, we used food intake entropy to quantify the mathematical unpredictability and fragmentation of under differing conditions (Fig. 3L). Consistent with the stability analysis, a two-way ANOVA on entropy values revealed significant main effects for feeding paradigm (*F*(2, 100) = 188.8, *p* < 0.0001) and light cycle (*F*(1, 100) = 20.22, *p* < 0.0001), accompanied by a significant interaction (*F*(2, 100) = 4.09, *p* = 0.0196). CD *ad libitum* mice exhibited significantly higher entropy than LD controls (Δ -0.47, *p* < 0.0001), representing a profound loss of behavioral predictability that closely mirrors their poor metabolic outcomes. In contrast, the CD TR Night paradigm served as a powerful stabilizer; the CD cycle did not significantly increase entropy in CD TR Night animals compared to their LD counterparts (*p* = 0.2445). These results demonstrate that time-restricted feeding shields the organism from the destabilizing effects of light-cycle shifts by maintaining a rigid, predictable, and low-entropy behavioral structure.

## Discussion

Our study reveals that disrupting the light-dark cycle (CD), by inducing a 6 h phase advance, every 6 days, critically determines metabolic phenotype by altering the temporal organization of food intake. In the absence of temporal feeding restrictions, CD exposure led to a progressive increase in light-phase food intake and a disruption to the regularity of the diurnal feeding rhythm. This resulted in increased body weight and higher body fat, despite no significant alterations in total caloric intake or voluntary wheel running activity compared to LD *ad libitum* animals.

Our observations corroborate previous findings from our laboratory^36^ and others^10,11,38–44^, and strongly suggest that mistiming of energy intake relative to the internal circadian clock is an independent and potent contributor to weight gain and adiposity. Others have shown that male mice increase body weight under chronic jet lag, whereas female mice lose weight^40,45^. This highlights an important sexual dimorphism in mice, in their response to chronic jetlag, and one that we were not able to study further due to our exclusive use of male mice. Recent evidence suggests that estrogen receptor-alpha signaling allows female mice a sharper re-entrainment angle to light/dark cycles following a phase shift, likely leading to a reduction in the length of internal desynchrony. Furthermore, estrogen signaling modulates the hypothalamic-pituitary-adrenal (HPA) axis, an essential mechanism of an organism's response to metabolic stress^46^. In the context of our experimental paradigm, the chronic 6-h phase advances serve as a recurring environmental stressor that repeatedly activates the HPA axis. In males, this chronic activation has been shown to correlate with increased glucocorticoid signaling that promotes metabolic efficiency and lipid storage under conditions of desynchrony^47^. However, the known modulation of the HPA axis by estrogen suggests a different trajectory for females. Estrogen is known to enhance the sensitivity of the HPA axis to stressors, which, during repeated light-cycle shifts, may favor a state of stress-induced hypophagia and increased non-shivering thermogenesis.

Interestingly, synchronizing a 12-h window of food intake with the disrupted light cycle (CD TR Night) abrogated the metabolic phenotype we observed in the CD *ad libitum* group. This was achieved by restricting food access to a 12 h window that tracked the shifting light-dark cycle. While this group displayed a modest reduction in total caloric intake compared to *ad libitum* controls in the later stages of the experiment, intake remained statistically identical between the LD and CD light cycles within each feeding paradigm. The most critical observation is that CD *ad libitum* mice exhibited significantly greater adiposity than LD *ad libitum* mice, despite maintaining nearly identical caloric intake (Figs. 2E, F and 3C, D). This divergence demonstrates that the temporal mismatch of intake relative to the light cycle, rather than total caloric volume, is the primary driver of the observed adipogenic phenotype. When food intake becomes misaligned and spread across the 24 h cycle, as seen in the CD *ad libitum* group, the body’s ability to temporally partition nutrients effectively is likely compromised, favoring lipid storage over oxidation. The fragmented feeding architecture and high entropy observed in CD *ad libitum* mice suggest a state of chronic internal desynchrony, where the absence of a clear “fasting signal” likely prevents the metabolic switch to lipid oxidation^48^. Based on previous research^21^, we predict that the 12-h shift in food availability in the CD TR Night group likely induced a corresponding 12-h phase shift in peripheral tissue clocks. By imposing this restriction, it’s possible that we restored a high-amplitude rhythm in metabolic regulators (e.g., PPAR*α*, PGC1*α*), effectively forcing the mice to switch between carbohydrate utilization and lipid mobilization, regardless of the shifting environmental light^49,50^. Furthermore, if the ‘rescue’ in the CD TR Night group were simply a byproduct of reduced intake (relative to CD *ad libitum*), one would still expect these animals to show some metabolic penalty from the shifting light cycle when compared to the LD TR Night group. Instead, the CD TR Night mice maintained body composition trajectories that were indistinguishable from their LD counterparts (Fig. 2). This provides evidence that temporal consolidation serves as a dominant metabolic stabilizer that can override the destabilizing effects of light-cycle disruption.

These results are consistent with the literature demonstrating that TRF mitigates metabolic issues induced by light and circadian disruptions. For example, restricting food access to the 12 h active period can rescue mice that gain weight under dim light at night^25^. Similarly, a 2021 study and additional follow-up study showed that TRF during chronic jetlag reduced body weight gain and restored rhythmic gene expression in the hypothalamus and jejunum, impacting nutrient transport and lipid metabolism^38,43^. Moreover, TRF offers metabolic protection even in mice with inherent molecular clock disruptions, guarding against high-fat diet-induced obesity^51^. Others have shown that, as an alternative to TRF, modulating light levels can be used to prevent circadian misalignment in mouse models of shiftwork^10,52^. These findings underscore the potent and conserved protective effect of aligning food intake with the appropriate active phase.

The unique novelty of our approach is best demonstrated by the LD TR Moving group. By introducing unpredictability in food availability into a standard light environment, we show that the loss of feeding regularity alone, independent of light cycle disruption, is enough to cause energy imbalance. Rather than interpreting this result as a benefit caused by circadian misalignment, the lean phenotype in these mice likely stems from the unpredictability of the food intake window. This instability may disrupt anticipatory feeding mechanisms and digestive efficiencies, ultimately leading to a net reduction in caloric intake. This observation, alongside the reduced food intake in the constantly shifting but aligned CD TR Night group, raises an intriguing question: Is the predictability of a diet more critical than the alignment of the diet with the circadian system as a whole? Our data suggest that unpredictable or constantly changing feeding schedules may disrupt the physiological processes that regulate appetite and satiety, leading to a net reduction in caloric intake and consequently, less weight gain. However, we are unable to determine the long-term effects of this desynchrony due to the short time frame of our study. This warrants further investigation into the behavioral and metabolic mechanisms underlying the impact of feeding predictability on energy balance and long-term health outcomes.

Our findings also highlight the differential influence of light and food on distinct physiological outputs. In all CD cycle groups, we observed a dramatic alteration in running wheel activity patterns, mirroring the shifts in the light-dark cycle. In stark contrast, disrupting food access under a stable light-dark cycle (LD TR Moving) had no discernible effect on the overall amount of running wheel activity. This underscores the dominant role of light as the primary zeitgeber for locomotor activity, typically mediated by the SCN master pacemaker. However, lean phenotypes observed in our restricted feeding groups, even when misaligned with the light cycle, point to the influence of the Food Entrainable Oscillator (FEO)^53^. Unlike the SCN, which is primarily entrained by light, the FEO is a distinct circadian mechanism that enables animals to anticipate and synchronize physiological and behavioral processes to food availability^54^. While the anatomical location of the FEO remains a subject of debate, its ability to function independently of the SCN creates a dual-oscillator system within the organism. In our model, the robustness of the FEO likely contributes to the inability of the LD TR Moving and CD TR Locked to predict mealtimes, leading to a marked reduction in caloric intake. In these two paradigms, it’s possible that we are moving the predictable window of food intake at a rate at which the FEO cannot keep up.

Finally, the observation that combined light and food disruption (CD TR Locked) did not exacerbate the effects of the singular disruptions alone provides insights into the relative strengths of these independent *zeitgebers*. The fact that the metabolic outcomes in the CD TR Locked group were similar to the CD TR Night group suggests that the mere act of an imposed feeding/fasting window serves as a dominant metabolic stabilizer on a short-term basis. While the shifting light cycle exerts a dominant influence over locomotor behavior, imposing a fixed feeding window locked to the initial LD cycle does not worsen the metabolic profile beyond that induced by the light disruption alone. This implies a functional hierarchy where light dictates the timing of activity, yet the temporal consolidation and amount of nutrient intake remain a superior determinant of adiposity in the short-term. Our data indicate that both consistent (CD TR Locked) and shift-aligned (CD TR Night) time-restricted feeding schedules offer significant metabolic protection compared to fragmented CD *ad libitum* intake. It remains to be seen whether the misalignment of food intake in the CD TR Locked group would lead to any negative health outcomes in the long-term, based on the results of this study.

Although this study successfully assessed the interaction of light cycle disruptions and nutrient timing on body composition, we acknowledge that this is only one facet of a broader metabolic profile. Because we did not directly measure energy expenditure, oxygen consumption, or glucose disposal rates, we cannot rule out the possibility that shifting light cycles alters metabolic rate or digestive efficiency in ways not captured by pellet counts and MRI. Future research must incorporate comprehensive metabolic phenotyping, including indirect calorimetry and detailed assessment of glucose and lipid metabolism, to fully elucidate the mechanisms underlying the observed effects of disrupted light and food zeitgebers. Furthermore, determining the long-term consequences of these circadian disruption paradigms and exploring therapeutic interventions targeting the circadian system and feeding behavior remain crucial next steps. Ultimately, this work suggests that while any form of temporal consolidation of food intake provides short-term metabolic protection against light-cycle disruption, it remains unseen as to the long-term negative impact that a “locked” feeding window may have. This implies that for shift workers, synchronizing nutrient intake with the biologically active period, rather than rigidly adhering to a static, misaligned schedule, may be a more effective strategy for maintaining long-term metabolic health.

## Methods

### Animals

C57BL/6J male mice (*n* = 108) were raised at the University of Texas Southwestern Medical Center (UTSW) in-house breeding colony (Mouse Breeding Core, Wakeland lab, UTSW, Dallas, TX, USA). Only male mice were used for this study to reduce potential variability associated with the estrous cycle and to establish a baseline metabolic response to the chronic shifting protocol. Animals were housed in a group housing facility (no more than 5 per cage) until the approximate age of 12 weeks, before being transferred into individual Tecniplast isolation cages (Catalog 1144B, 33.11 *×* 15.9 *×* 15.0 cm (852 cm^3^), Tecniplast, Italy) with a single stainless steel running wheel (12.0 cm in diameter). Temperature and humidity levels were monitored throughout the experiment and maintained between 20–27°C and 30–70% relative humidity. The Institutional Animal Care and Use Committee (IACUC) of the University of Texas Southwestern Medical Center approved the animal protocol (Animal Protocol Number 2015-100925).

### Lighting and feeding conditions

Animals were maintained under either a 12-h light or 12-h dark cycle (Control; LD Cycle) (Fig. 1A), or a 12:12 light/dark cycle that was advanced by 6 h every 6 days (Chronic Disruption; CD Cycle) (Fig. 1B). During lights on, green LEDs provided ~100 lux at the level of the cage floor. Green LEDs were selected to provide a narrow-spectrum light source (peak ~510 nm) that aligns with the peak sensitivity of the mouse circadian system, specifically targeting melanopsin-containing retinal ganglion cells and M-cones, ensuring efficient entrainment while minimizing potential non-circadian effects associated with broad-spectrum white light^55^. On the day of the advance, the 12-h dark period was shortened to 6 h, leading to a phase advance in the normal 12:12 light/dark cycle. Food access and food recordings were conducted by specialized feeding equipment developed in-lab with the assistance of Phenome Technologies Inc., Skokie, IL, USA^56^. Mice were fed round pellets of 315 ± 4 mg each containing 3.65 Kcal/g (Dustless Precision Pellets, Rodent, Grain-Based, F0170, BioServ, Flemington, NJ, USA). Pellet composition is similar to regular mouse chow (21.3% protein, 3.8% fat, 54.0% carbohydrates).

Following the transfer of animals from grouped housing to individual cages at the age of 12 weeks, all groups were fed *ad libitum* for the first week, to allow animals to become accustomed to the food delivery system. Following this, animals were weight-matched and randomly assigned to differing feeding regimes. Animals were fed either *ad libitum* (LD *adlibitum*, *n* = 24; CD *ad libitum*, *n* = 12), had food access restricted to the 12-h period during lights off (LD TR Night, *n* = 24; CD TR Night, *n* = 12), had a 12 h window of food access shifted by 6 h every 6 days (LD TR Moving, *n* = 24), or had a 12 h window of food access that remained at a constant time, despite a shifting light/dark cycle (CD TR Locked, *n* = 12). In the case of the CD TR Night fed animals, access to food followed the light cycle shifts throughout the experiment, maintaining food access to the dark phase exclusively.

### Feeding and behavioral monitoring

Feeding behavior was monitored by an automated feeder system that controlled the duration, amount, and timing of food access. Automated feeding boxes work by dropping a single pellet of food from a food hopper, down a chute, leading to the base of a wire cage top, where mice have access to a single pellet. Here, a second sensor records when the mouse takes the pellet from the wire top. This setup meant that in some instances, food that had been dropped during the feeding window (e.g., at ZT 23:59 in TR Night fed animals) could potentially remain at the top of the cage, available to the mouse, for the entirety of the light phase, or until the mouse chose to take this final pellet. This meant that a single pellet could have been eaten during a time in which food access was turned off, in the case of the groups with restricted feeding times. This accounted for 7.56% in LD TR Night and 7.51% in CD TR Night of total food consumption. Issues arose in animals that displayed hoarding behavior, whereby animals would constantly take pellets from the pellet access point, without consuming them, and proceed to hoard them in their cage. This behavior was seen in less than 3% of animals and was corrected for by counting the number of pellets in their cage at the time of cage change, thereafter removing that amount from their total intake record. All included animals met the behavioral criteria for this study; specifically, no mice were excluded for food hoarding or off-schedule intake during restricted feeding windows.

Mice had access to running wheels within their own individual cage, which were constantly monitored to count wheel revolutions. Infrared sensors recorded every revolution of the running wheels, regardless of direction. This data was then collected using the Actimetrics Software, at a time resolution of 1 min (ClockLab, IL, USA). Both feeding activity and running wheel data were analyzed using RStudio software for R (RStudio, Boston, MA, USA) and Python programming.

### Body composition

To measure the body composition of the mice, we used an EchoMRI (Houston, TX, USA) machine, which is a non-invasive method that employs magnetic resonance imaging (MRI) technology. Measurements of all mice in the study were taken following the 15th phase advance, and 4 days prior to termination, at shift 25. Animals were first weighed, then secured within acrylic tubing before being placed inside the EchoMRI machine for measurement. Animals were scanned for ~30 s before being returned to their home cage. Body fat percentage was calculated against the body weight measurement on the day of recording.

### Statistical and reproducibility

Statistics were performed using a combination of GraphPad Prism Software, custom R scripts and custom Python scripts. Each individual mouse was treated as a biological replicate. Longitudinal data were analyzed by fitting a linear mixed-effects model, followed by a Type III three-way ANOVA using Wald chi-square tests to evaluate the effects of time, light cycle, and feeding paradigm. Post-hoc comparisons with Holm–Bonferroni adjustments were used to identify specific differences between groups across experimental time points. Prior to final model selection, visual inspection of residual plots was employed to confirm the assumption of homoscedasticity. To quantify the total impact of the intervention, the cumulative data were analyzed using a Type III two-way ANOVA, with light cycle and feeding schedule as the primary factors. Post-hoc pair-wise analysis using Holm-Šídák adjustments was used to determine differences between experimental groups. Autocorrelation was calculated by binning food intake data into 1-h bins and calculating the correlation of each day with every other day. Statistics were performed on the mean correlation coefficient of each individual animal by two-way ANOVA, and pairwise post-hoc analysis using Holm–Šídák adjustments. Data was visually inspected prior to parametric testing to confirm normality.

Entropy was calculated by utilizing Shannon’s Entropy, and quantifies the unpredictability or “surprise” inherent in daily food intake rhythms. A low entropy value signifies highly regular and predictable eating patterns, where meals consistently occur at expected times, reflecting a robust temporal organization of food intake. Conversely, high entropy indicates a less predictable and more variable rhythm, with eating events spread less consistently throughout the day, suggesting a greater “surprise” in when food consumption occurs. This metric offers a powerful way to objectively assess the regularity of an individual animal’s feeding patterns.

## Data Availability

The datasets generated and/or analyzed during the current study are not publicly available due to the datasets being part of an ongoing long-term research project and scheduled for further analysis in forthcoming publications but are available from the corresponding author on reasonable request.
